# The impact of seasonal temperature and water transport on the growth of sunshine rose grapevines and precision irrigation strategies

**DOI:** 10.3389/fpls.2025.1607731

**Published:** 2025-10-20

**Authors:** Ranran Wang, Zhen Han, Yingxiu Li, Suya Shang, Bo Li, Xin Lu

**Affiliations:** ^1^ College of Mechanical and Electronic Engineering, Shandong Agricultural University, Tai’an, China; ^2^ Engineering Laboratory of Agricultural Equipment Intelligence, Shandong Agricultural University, Tai’an, China; ^3^ Shandong Academy of Grape, Shandong Academy of Agricultural Science, Jinan, China; ^4^ Faculty of Science and Engineering, Iwate University, Morioka, Japan

**Keywords:** plant growth, water transport, temperature, transpiration, data-driven analysis, precision irrigation

## Abstract

This study explores the seasonal variations in grapevine growth and sap flow, with a particular focus on how environmental factors influence key growth indicators. Grapevines are highly sensitive to seasonal changes, and understanding these variations is essential for optimizing vineyard management practices. Given the increasing importance of precision agriculture, high-precision sensors were employed to monitor sap flow, leaf temperatures, and ambient temperature over the course of a year. By collecting data on these physiological indicators, we aim to identify patterns that can improve our understanding of grapevine responses to environmental changes. Our findings reveal significant seasonal fluctuations in grapevine growth, with the most growth occurring during the warmer months (spring and summer) and slower growth in winter. The comparison of predictive models, including Prophet, LightGBM, and XGBoost, demonstrated that machine learning models were more accurate in predicting grapevine growth compared to traditional methods. These results offer important insights into the relationship between grapevine physiology and environmental conditions, providing a foundation for improving vineyard management practices. The grape variety utilized in this study is Sunshine Rose (*Shine Muscat*), known for its distinctive sweet flavor and high economic value, making it a popular cultivar in vineyards worldwide.

## 1 Introduction

Grape cultivation plays a vital role in global agriculture, particularly in the wine and table grape industries. With the escalating challenges of climate change and water resource shortages, effective water management has become a crucial aspect of sustainable grape cultivation. The interactions between temperature, water transport, and grapevine growth are central to grape physiology research. Variations in temperature directly impact transpiration, water transport, and nutrient absorption, which, in turn, influence growth and fruit quality. Although the adoption of precision irrigation and digital agriculture technologies offers promising solutions, there remains a gap in current research, particularly in developing precision irrigation strategies that are driven by multi-parameter sensor data. This study aims to address this gap by exploring the seasonal variations in grapevine growth and sap flow and examining how environmental factors, monitored through high-precision sensors, influence water management strategies and grapevine development.

Recent studies confirm that soil water availability strongly regulates grapevine transpiration and water-use efficiency, as shown by multi-sensor assessments combining sap flow, leaf gas exchange and chlorophyll fluorescence (Benyahia et al., 2023). Furthermore, the impact of temperature on grapevine transpiration cannot be overlooked. Under drought conditions, the nitrate absorption rate decreases and is closely linked to changes in water status (Gloser et al., 2020). Leaf temperature monitoring has also been widely adopted as a direct proxy of canopy transpiration and stress responses (Zhou et al., 2022). These environmental interactions collectively determine the efficiency of grapevine growth and water transport. Improved transpiration models offer more accurate predictions of crop transpiration rates (Choi and Shin, 2020).

The relationship between transpiration and nutrient absorption is a major focus of plant physiology research. Vineyard studies in the last decade demonstrated that canopy conductance and transpiration 40 under water stress strongly regulate nutrient uptake efficiency, with hysteresis patterns highlighting the dynamic coupling of physiology and environment (Bai et al., 2015). Nitrogen affects water flow and nutrient absorption by regulating stomatal conductance and root hydraulic conductivity (Matimati et al., 2014). Nighttime transpiration may promote leaf nutrient absorption, especially under phosphorus-deficient conditions (Vega et al., 2023), and earlier work suggested that transpiration-driven mass flow can enhance nutrient transport to the roots (Cramer et al., 2008). Sensor-fusion approaches further indicate that grapevines dynamically adjust water and nutrient uptake pathways under stress, which can be captured by real-time monitoring of stem water potential (Ohana-Levi et al., 2022). Emerging approaches highlight the potential of integrating multi-sensor data streams, such as thermal, VNIR and RGB imagery, to refine vineyard water-stress detection and irrigation decision-making (Burchard-Levine et al., 2024).

With the development of data-driven and machine-learning methods, researchers are now able to more accurately predict plant growth dynamics and vineyard water requirements. Machine-learning approaches that integrate environmental and physiological data have emphasized the interaction between water availability and nutrient uptake, offering flexible alternatives to traditional mechanistic models (Fuentes et al., 2024). Multi-sensor and proximal-sensing frameworks that combine thermal and VNIR/multispectral imagery with weather inputs have demonstrated strong potential for detecting vine water stress and improving prediction accuracy (Tang et al., 2022). At regional scales, remote sensing combined with machine learning has been applied to map irrigated vineyard areas and support large-scale irrigation planning (López-Pérez et al., 2024). In addition, sap-flow-based modeling continues to capture grapevine transpiration responses to environmental drivers, providing a physiological foundation for precision irrigation strategies (Wei et al., 2020).

Improving water-use efficiency (WUE) in precision viticulture is closely tied to phenology-aware management of canopy transpiration. At the plot scale, the dual crop-coefficient (dual-Kc) approach has increased water productivity in *Vitis vinifera* cv. Alvarinho, indicating more efficient allocation of irrigation across growth stages (Silva et al., 2024). In a three-season field study with Cabernet Sauvignon, data-driven irrigation scheduling based on ET*_c_* and plant/soil water-status thresholds reduced applied water by up to 65% while increasing crop-level WUE by as much as 41% on lighter soils, without clear yield penalties (Schlank et al., 2024). Complementarily, decision-support systems for precision regulated deficit irrigation that predict soil moisture and recommend schedules have demonstrated practical feasibility in vineyard settings (Kang et al., 2023). In line with this perspective, the present study focuses on the temperature–water-transport–growth axis, with nutrient-uptake aspects referenced only as background.

Mineral nutrition interacts with plant water transport but is not the focus here. Long-distance nutrient delivery emerges from transpiration-driven mass flow (e.g., nitrate) and diffusion (e.g., phosphate), with their contributions depending on nutrient form and soil supply (Plett et al., 2020; Holz et al., 2024). In grapevine, nutrient status can feed back on water fluxes—for example, potassium deficiency reduces transpiration via decreases in leaf area and stomatal conductance (Sperling et al., 2024). In this study, nutrient aspects are referenced only as contextual background to the temperature–water-transport–growth axis.

Precision irrigation in vineyards has shifted from rule-based fertigation toward sensing- and model-driven scheduling. Contemporary reviews and field implementations show that decision-support systems fuse plant/soil/atmospheric sensing with meteorological inputs to recommend irrigation timing and amounts, improving operational efficiency and water savings (Tardaguila et al., 2021). In commercial settings, both a CWSI-based IoT DSS and a soil-moisture-driven DSS have been deployed over multiple seasons, maintaining yield and quality while reducing applied water (e.g., 10–17%) (King and Shellie, 2023; Garofalo et al., 2023).

This study integrates multi-parameter sensing with high-frequency modeling to translate physiological dynamics into phenology-aware irrigation guidance. Vines were instrumented with high-precision sensors to continuously monitor fruiting cane diameter (DC1), one-sided cordon diameter (DC2), trunk sap flow, leaf temperature (Leaf1–Leaf3), and ambient temperature. Seasonal forecasting pipelines paired tree-based ensembles (LightGBM, XGBoost) with an explicit seasonal component (Prophet). Combined with vertical profiling (cane–cordon–trunk) and vertical-gradient analysis, the models reveal spatiotemporal patterns of water transport and radial growth and yield decision-oriented thresholds for irrigation timing and amount. It was hypothesized that seasonal fluctuations in environmental temperature modulate grapevine water transport and radial growth, and that machine-learning models can predict these dynamics with actionable accuracy. Specifically, the aims were to: (i) quantify cross-season relationships among environmental temperature, sap flow, leaf temperature, and radial growth; (ii) evaluate forecasting performance from high-frequency signals; and (iii) propose phenology-aware, sensor-based irrigation guidelines.

## 2 Materials and methods

### 2.1 Experimental materials and site overview

**Figure 1:**
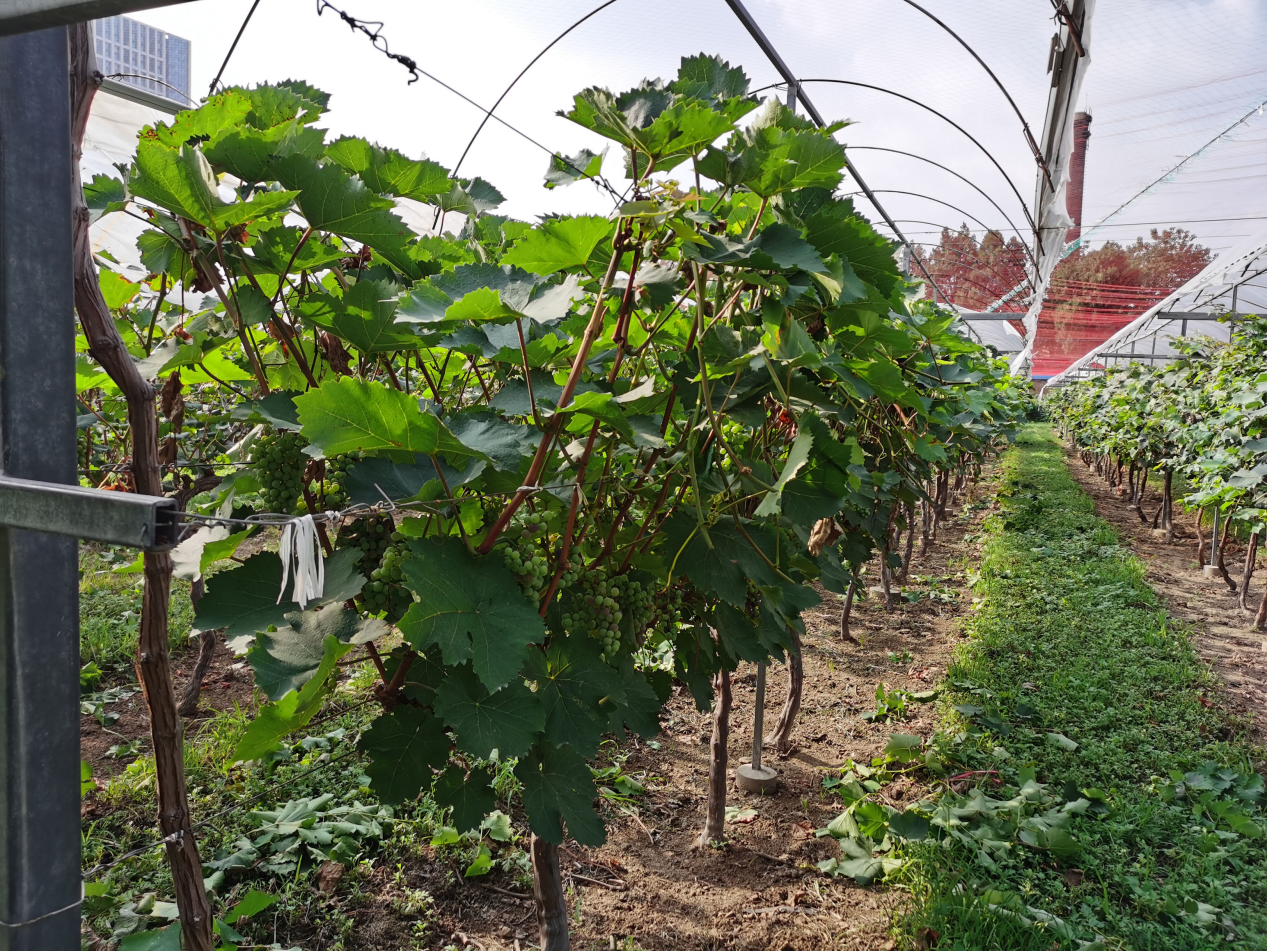
Single-trunk double-arm V-shaped trellis grapevines at the Jinniushan Grape Experimental Base.

The grape variety used in this study was *Sunshine Rose*, a high-value cultivar known for its distinctive sweet flavor. Vines were trained to a single-trunk, double-arm V-shaped trellis to maximize light interception, control canopy architecture, and promote uniform ripening (**Figure 1**). Vines were 10 years old at the start of monitoring. The experimental block comprised 96 vines arranged in six north–south oriented rows (16 vines per row) at 2.5 m (row) × 1.2 m (vine) spacing; twelve representative vines were instrumented, and the first and last rows were reserved as buffers. Irrigation was supplied via a pressure-compensating drip system (two laterals per row; emitters 2.0 Lh^−1^ at 0.5 m spacing; operating pressure 0.2 MPa) with Venturi-based fertigation every 10–14 days during the growing season.

The study was conducted in a plastic greenhouse at the Jinniushan Grape Experimental Base, Tai’an, Shandong, China (temperate monsoon climate). Greenhouse climate was controlled with automated shading and ventilation; relative humidity was maintained at 60–70% through humidification and misting as needed. The soil was sandy loam (pH 6.5–7.0) with good drainage and aeration. Soil nutrient status was monitored regularly, and fertigation was adjusted accordingly to ensure balanced nutrition throughout the growth cycle.

### 2.2 Sensor configuration and data collection

We used high-precision sensors (Dynamax Inc., Houston, TX, USA) to continuously monitor the growth environment and physiological status of *Sunshine Rose* grapevines. The configuration comprised: (i) two DEX dendrometers for branch diameter dynamics (DC1 on the fruiting cane; DC2 on the one-sided cordon); (ii) three SapIP-IRT wireless infrared sensors for leaf-surface temperature (Leaf1–Leaf3); (iii) one SapIP trunk sap-flow probe (TDP1); and (iv) one ambient-temperature probe at canopy height. All sensors were factory-calibrated; zero-offset and drift checks were performed before deployment and during routine maintenance. Data were logged hourly from 1 June 2020 to 1 June 2021 (UTC + 8), yielding 8,760 records per channel (61,320 channel-hours across the seven channels). Key phenological stages (budburst, flowering, veraison, maturity) were recorded and used to align physiological signals (e.g., sap flow, radial variation) with vine development for stage-aware analyses.

**Figure 2:**
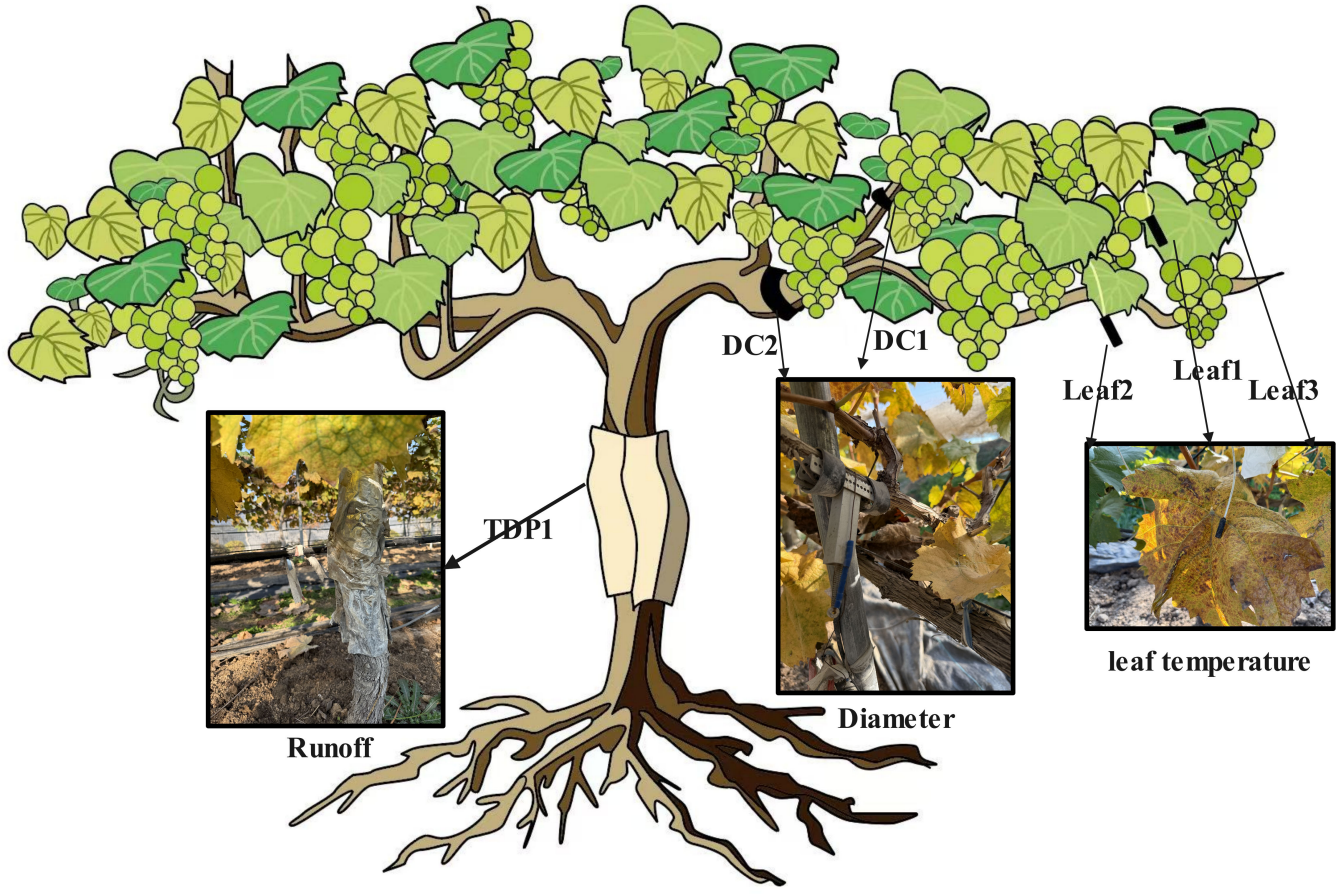
Installation positions of the sensors.

Sensor locations were standardized to ensure both vertical and organ-level coverage (**Figure 2**). DC1: fruiting cane, ∼15 cm from the node with the cordon (avoiding junctional artifacts while capturing typical cane growth). DC2: one-sided cordon, ∼35 cm from the trunk node (minimizing trunk influence on cordon diameter). TDP1: trunk, ∼60 cm above ground (integrative measure of whole-plant water transport). Leaf1: mid-canopy on the fruiting cane; Leaf2: upper canopy on the one-sided cordon; Leaf3: upper canopy on the fruiting cane. This layout captured vertical gradients (mid vs. upper canopy) and organ differences (cane–cordon–trunk) while maintaining representativeness across the canopy.

Hourly monitoring across summer (June–August), autumn (September–November), winter (December–February), and spring (March–May) enabled the capture of short-term fluctuations and cross season shifts in environmental drivers, providing high-resolution inputs for subsequent modeling of growth and water-transport dynamics.

### 2.3 Data preprocessing

During data collection, a comprehensive data cleaning process was implemented to ensure high data quality and improve analysis accuracy. The Z-score method was applied to remove invalid or abnormal data points, such as extreme temperature values or measurements affected by sensor malfunctions or external disturbances, ensuring that outliers did not compromise the dataset’s reliability. Missing values were addressed through linear interpolation, where data points that were missing or unrecorded were estimated based on the surrounding valid data, effectively filling gaps and maintaining continuity in the time series. Time synchronization was also performed to resolve discrepancies in timing across sensors. All sensor data were aligned to a unified timestamp, ensuring consistency across measurements taken at different times. Furthermore, unit conversion and normalization were applied to standardize the data from different sensors with varying units or scales, allowing for direct comparison and analysis on a uniform scale. These preprocessing steps ensured the accuracy, consistency, and completeness of the dataset, providing a solid foundation for reliable statistical analysis and modeling.

### 2.4 Data analysis methods

This study employed multiple data analysis methods to explore the relationships between temperature, water, and physiological indicators during grapevine growth and to construct predictive models. Pearson correlation coefficients were calculated to quantify the correlations between environmental temperature, sap flow, trunk radial growth, and leaf temperature. The results were visualized using heatmaps to intuitively reflect the positive and negative correlations among the indicators. The trends of different indicators across seasons were compared to reveal the mechanisms by which environmental factors influence grapevine growth. The results were presented in charts, reflecting the seasonal variation patterns of the indicators.

LightGBM and XGBoost were selected due to their strong performance on small tabular datasets and their robustness in handling missing or noisy data. Prophet was chosen for its ability to explicitly model seasonal trends. In this study, linear regression, LightGBM, XGBoost, and Prophet models were constructed using leaf temperature, trunk sap flow, and environmental temperature as input features, with grapevine diameter as the target variable. Model performance was evaluated using metrics including MSE, RMSE, MAE, MAPE, and R². The results demonstrated that LightGBM and XGBoost outperformed linear regression in capturing nonlinear relationships, while Prophet effectively modeled seasonal growth patterns. Future studies will consider incorporating additional environmental variables (e.g., soil moisture, light intensity, meteorological data) and exploring deep learning approaches such as LSTM or Bi-LSTM to further enhance prediction accuracy and capture long-term temporal dependencies in physiological signals.

Visualization methods such as heatmaps, line charts, and scatter plots were used to clearly present the analysis results, facilitating the understanding of complex relationships among indicators and their seasonal variations.

## 3 Results

### 3.1 Overall dynamics of the temperature-water-growth system

**Figure 3:**
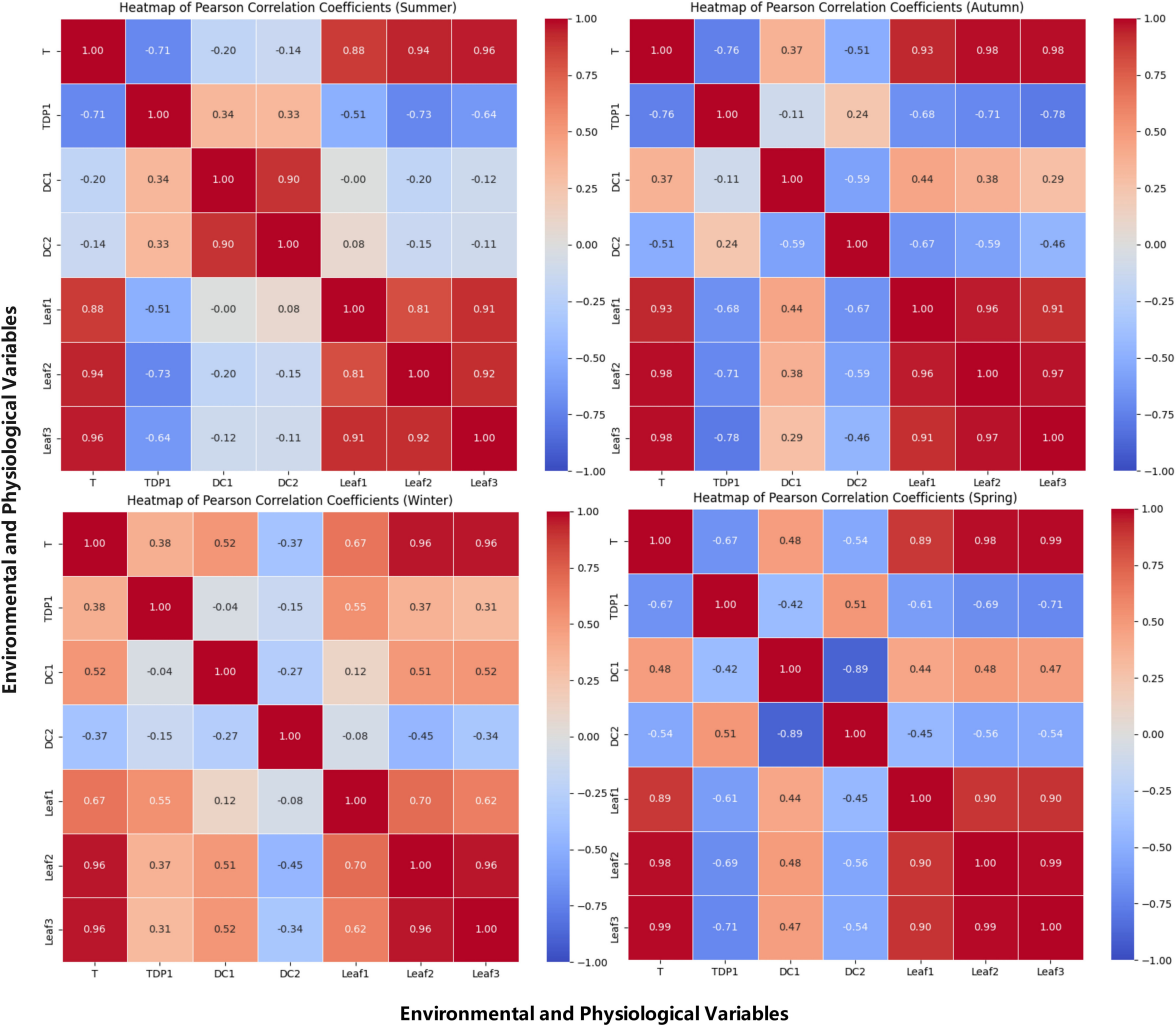
Pearson correlation heatmap of temperature and physiological indicators of grapevines. This figure presents a Pearson correlation heatmap illustrating the relationships between ambient temperature, leaf temperatures (Leaf1, Leaf2, Leaf3), and physiological indicators of grapevines, including sap flow (TDP1) and grapevine diameter (DC1, DC2). The heatmap highlights the strength and direction of the correlations, with warmer colors indicating a strong positive correlation and cooler colors showing a negative correlation. This visual representation helps to identify key environmental factors that influence grapevine growth and physiological processes across different seasons.

The spatiotemporal dynamics of environmental factors during grape growth were revealed by analyzing the correlations between environmental temperature, water status, and grapevine growth across seasons. Pearson heatmaps quantified relationships among ambient temperature, trunk sap flow rate, stem diameter, and leaf temperature, visually illustrating the strength and direction of these correlations across seasons. Specifically, the Pearson correlation heatmaps for spring, summer, autumn, and winter are shown in **Figure 3**. These heatmaps are descriptive screens to locate season- and organ-specific coupling; mechanistic interpretation and implications are addressed in the Discussion.

#### 3.1.1 Seasonal analysis of the relationship between canopy temperature and trunk sap flow rate

The relationship between temperature and trunk sap flow rate was observed to exhibit significant seasonal variations, reflecting the physiological responses of grapevines to temperature changes. The specific seasonal analyses are as follows:

Summer: A strong negative correlation was observed (r = –0.71), consistent with stomatal regulation under sustained heat and high atmospheric demand.

Autumn: The negative correlation (r = –0.76) persisted as canopy activity declined, indicating demand–supply decoupling during senescence.

Winter: The positive correlation (r = 0.38) likely reflects warmer intervals reducing sap viscosity and thawing conductive pathways, allowing modest increases from low baselines.

Spring: The negative correlation (r = –0.67) suggests transient heat spells depress conductance during early growth.

#### 3.1.2 Seasonal characteristics of fruiting cane diameter and one-sided cordon diameter

**Figure 4:**
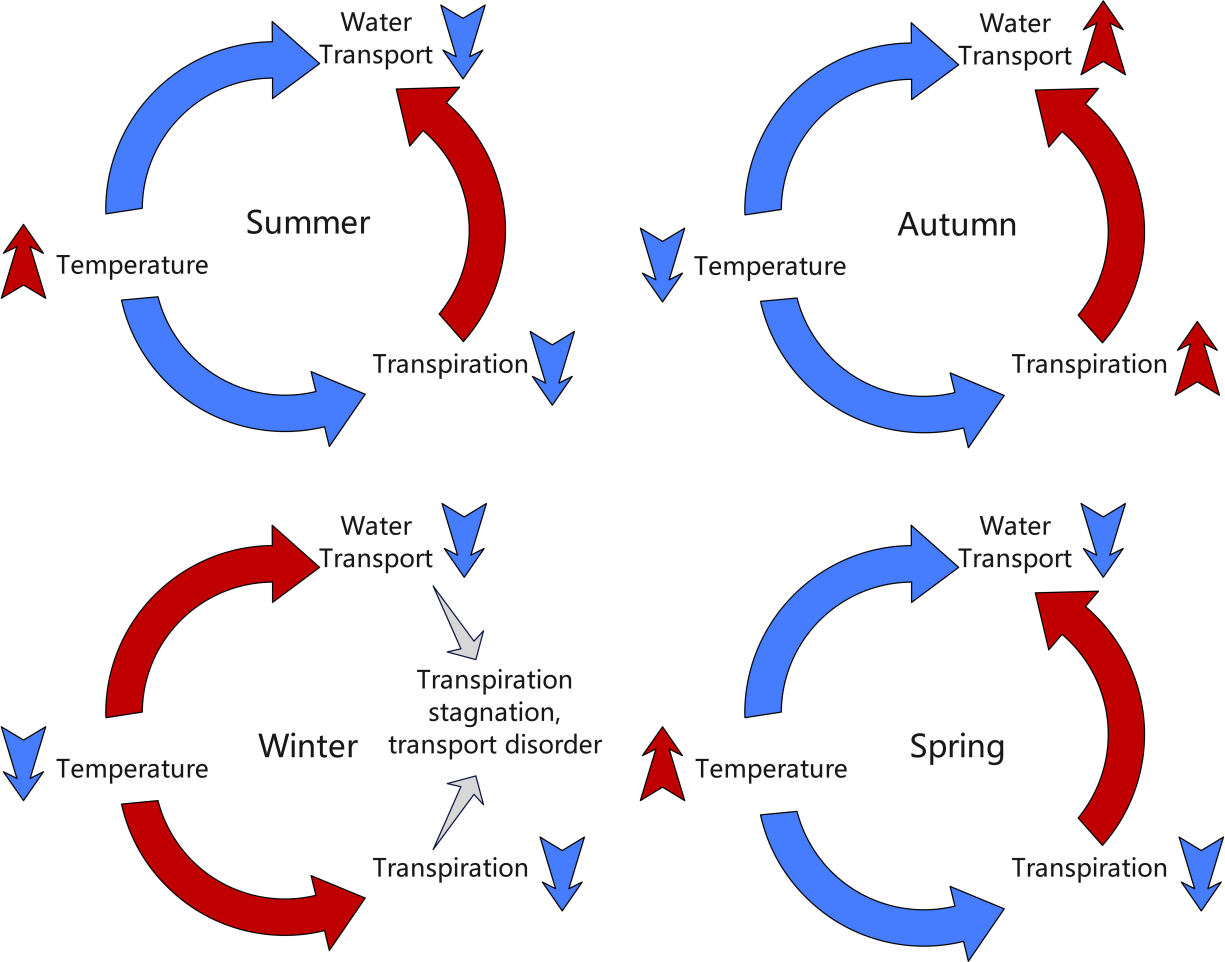
Hypothesized seasonal interactions between temperature, transpiration, and water transport in grapevines. The diagram illustrates how these environmental factors influence grapevine growth dynamics across different seasons (spring, summer, autumn, and winter).

Significant seasonal variations were observed in the radial growth synchronization between different parts of the grapevine, reflecting the profound impact of environmental conditions on grapevine growth activities. To provide a more intuitive understanding of the seasonal interactions between temperature, transpiration, and water transport, **Figure 4** illustrates how these factors influence grapevine growth dynamics across different seasons.

Summer: The correlation coefficient between DC1 and DC2 was 0.9, indicating a positive correlation. High temperatures and abundant sunlight provided optimal growing conditions for the grapevine, leading to significantly enhanced overall growth activity. This high correlation reflected the highly synchronized radial growth trends of the grapevine under warm conditions. With favorable temperatures and ample light, the grapevine experienced vigorous growth, resulting in a substantial increase in growth synchronization between the two parts, manifested as a strong positive correlation.

Autumn: As temperatures began to drop, the correlation coefficient between DC1 and DC2 decreased to –0.59, showing a negative correlation. The grapevine’s water use efficiency declined, evaporation decreased, and growth activity began to slow. This negative correlation suggested that, as the temperature decreased, the growth trends of the two parts became divergent. This divergence may be attributed to differences in how each part responded to temperature changes, reflecting the gradual weakening and inconsistency of grapevine growth activities during autumn.

Winter: The correlation coefficient between DC1 and DC2 further decreased to –0.27, indicating a weak negative correlation. Under low temperatures, the grapevine entered a nearly dormant state, and overall growth activity significantly decreased. This weak negative correlation reflected more noticeable growth differences between the different parts of the trunk during dormancy. However, as the temperature’s suppressive effect on growth was relatively uniform, the negative correlation between the two parts was weakened.

Spring: The correlation coefficient between DC1 and DC2 further dropped to –0.89, indicating a significant negative correlation. As temperatures rose, the grapevine gradually resumed growth activity, but the growth differences between the two parts became more pronounced. This strong negative correlation suggested that as temperatures increased, faster water evaporation intensified competition for water and nutrients among the different parts, leading to greater asynchrony in growth between the DC1 and. Consequently, the negative correlation between the two parts significantly strengthened.

#### 3.1.3 Spatiotemporal dynamics of water transport vertical gradient (and upper-lower water association

Significant seasonal variations in the correlations between diameter and trunk sap flow rate were observed, reflecting differences in physiological activities under varying environmental conditions.

Summer: Positive correlations were observed between DC2 and TDP1, with a correlation coefficient of 0.33, and between DC1 and TDP1, with a correlation coefficient of 0.34. These positive correlations indicated that water demand and flow were coordinated across different parts of the grapevine under high temperatures. The vigorous growth activity during summer was found to ensure strong synchronization in the vertical gradient of water transport and the upper–lower water association.

Autumn: The correlation coefficient between DC2 and TDP1 was reduced to 0.24, indicating a weakened positive correlation, while a weak negative correlation was observed between DC1 and TDP1, with a correlation coefficient of –0.11. This suggests that water transport became less synchronized as temperatures dropped and grapevine growth slowed. The decrease in evaporation and slower growth led to divergent water demand across different parts of the grapevine, causing inconsistencies in the vertical gradient and upper–lower water association.

Winter: A significant negative correlation was observed between DC2 and TDP1, with a correlation coefficient of –0.51, while a very weak negative correlation was found between DC1 and TDP1, with a correlation coefficient of –0.04. Under low temperatures, grapevines were observed to enter a near-dormant state, and water evaporation was significantly reduced, leading to asynchronous water transport. This negative correlation indicated the near-complete cessation of growth in the grapevine during dormancy, leading to stagnation in water transport. Significant differences were observed in the vertical gradient and upper–lower water association.

Spring: A positive correlation was observed between DC2 and TDP1, with a correlation coefficient of 0.51, while a negative correlation was found between DC1 and TDP1, with a correlation coefficient of –0.42. This suggested that water transport in the thicker one-sided cordon became more synchronized with trunk sap flow, while the fruiting cane exhibited asynchronous water demand. As temperatures rose, grapevines were observed to gradually resume growth, and water transport became more active. However, the competition for water and nutrients among different parts was found to intensify, leading to a divergence in water transport synchronization. This reflected the dynamic spatiotemporal changes in the vertical gradient and upper–lower water association during the spring recovery period.

### 3.2 System association between environmental temperature and leaf temperature

The relationship between environmental temperature and leaf temperature exhibited significant seasonal variations, reflecting the response mechanism of grapevine leaves to temperature changes.

Summer: A positive correlation between environmental temperature and leaf temperatures, with correlation coefficients between environmental temperature and Leaf1, Leaf2, and Leaf3 of 0.88, 0.94, and 0.96, respectively. This high positive correlation indicated that despite the generally high temperatures, the leaf temperatures followed a similar trend to the environmental temperature. However, as Leaf3 was more exposed to direct sunlight, it experienced more fluctuation in temperature than did Leaf1 and Leaf2, which were less exposed.

Autumn: The correlation between environmental temperature and leaf temperature remained high, with correlation coefficients between environmental temperature and Leaf1, Leaf2, and Leaf3 of 0.93, 0.98, and 0.98. This high positive correlation suggested that, despite the temperature decrease, the leaf temperatures across different parts of the grapevine still closely mirrored the environmental temperature, reflecting a uniform cooling effect across the plant.

Winter: The correlation between environmental temperature and leaf temperature remained relatively high. The correlation coefficients between environmental temperature and Leaf1, Leaf2, and Leaf3 were 0.70, 0.96, and 0.96, respectively. This positive correlation indicated that, under low temperatures, the leaf temperatures across different parts of the vine became more synchronized. However, Leaf1, positioned lower on the vine, experienced less variation in temperature compared to the more exposed leaves, which were more sensitive to the cold.

Spring: As temperatures began to rise in spring, a positive correlation between environmental temperature and leaf temperature, with correlation coefficients between environmental temperature and Leaf1, Leaf2, and Leaf3 of 0.96, 0.98, and 0.99. This high positive correlation suggested that as the temperature increased, the leaf temperatures across different parts of the grapevine became highly synchronized, reflecting a uniform warming effect on the entire vine.

### 3.3 The complex relationship between leaf temperature and trunk sap flow

Summer: Strong negative correlations were observed between Leaf1, Leaf2, and Leaf3 and TDP1, with correlation coefficients of –0.51, –0.73, and –0.64, respectively. Under high temperatures, transpiration in the grapevine peaked. This strong negative correlation was attributed to the significant enhancement of leaf transpiration under high temperature conditions, which led to a substantial reduction in trunk sap flow. The strongest negative correlation (–0.73) was found between the one-sided cordon Leaf2 and sap flow, likely due to its proximity to the trunk, where transpiration had a more direct and significant impact. The top fruiting cane Leaf3 also exhibited strong transpiration, with a slightly weaker negative correlation (–0.64) compared to Leaf2. The middle fruiting cane Leaf1, with relatively weaker transpiration, showed the weakest negative correlation (–0.51).

Autumn: As temperatures gradually decreased in autumn, the correlation coefficients between Leaf1, Leaf2, and Leaf3 and TDP1 were –0.68, –0.71, and -0.78, respectively, indicating negative correlations. Although grapevine growth slowed and transpiration weakened as temperatures dropped, the negative correlation persisted, suggesting that transpiration still contributed to a reduction in trunk sap flow. The top fruiting cane Leaf3 continued to exhibit significant transpiration, with the strongest negative correlation (–0.78). The one-sided cordon Leaf2, being closer to the trunk, still had a significant effect on sap flow, showing a strong negative correlation (–0.71). The middle fruiting cane Leaf1, with weaker transpiration, displayed the weakest negative correlation (–0.68).

Winter: The correlation coefficients between Leaf1, Leaf2, and Leaf3 and TDP1 were 0.55, 0.37, and 0.31, respectively, indicating positive correlations. At low temperatures, grapevines almost entered dormancy, and transpiration significantly decreased. This positive correlation was attributed to the reduction in leaf transpiration under low temperature conditions, leading to an increase in trunk sap flow. The middle fruiting cane Leaf1, found lower on the plant, was less affected by the cold and showed a stronger positive correlation (0.55) with sap flow. Both the one-sided cordon Leaf2 and the top fruiting cane Leaf3, being more exposed to the cold, showed relatively weaker positive correlations with sap flow (0.37 and 0.31, respectively).

Spring: As temperatures began to rise in spring, negative correlations were observed between Leaf1, Leaf2, and Leaf3 and TDP1, with correlation coefficients of –0.61, –0.69, and –0.71, respectively. With the rise in temperature and the resumption of growth activities, transpiration gradually intensified, leading to a reduction in trunk sap flow. The top fruiting cane Leaf3, exposed to stronger sunlight, exhibited more significant transpiration, resulting in a stronger negative correlation with sap flow (–0.71). Due to its proximity to the trunk, the one-sided cordon Leaf2 had a more direct impact on sap flow, showing a stronger negative correlation (–0.69). The middle fruiting cane Leaf1, with relatively weaker transpiration, displayed the weakest negative correlation (–0.61).

The seasonal variation in correlations highlights the profound impact of temperature-driven transpiration on water transport in grapevines, reflecting differences in the response of leaves to temperature changes at various locations on the vine and illustrating the grapevine’s physiological adaptation mechanisms under different environmental conditions.

### 3.4 Granger causality test

**Figure 5:**
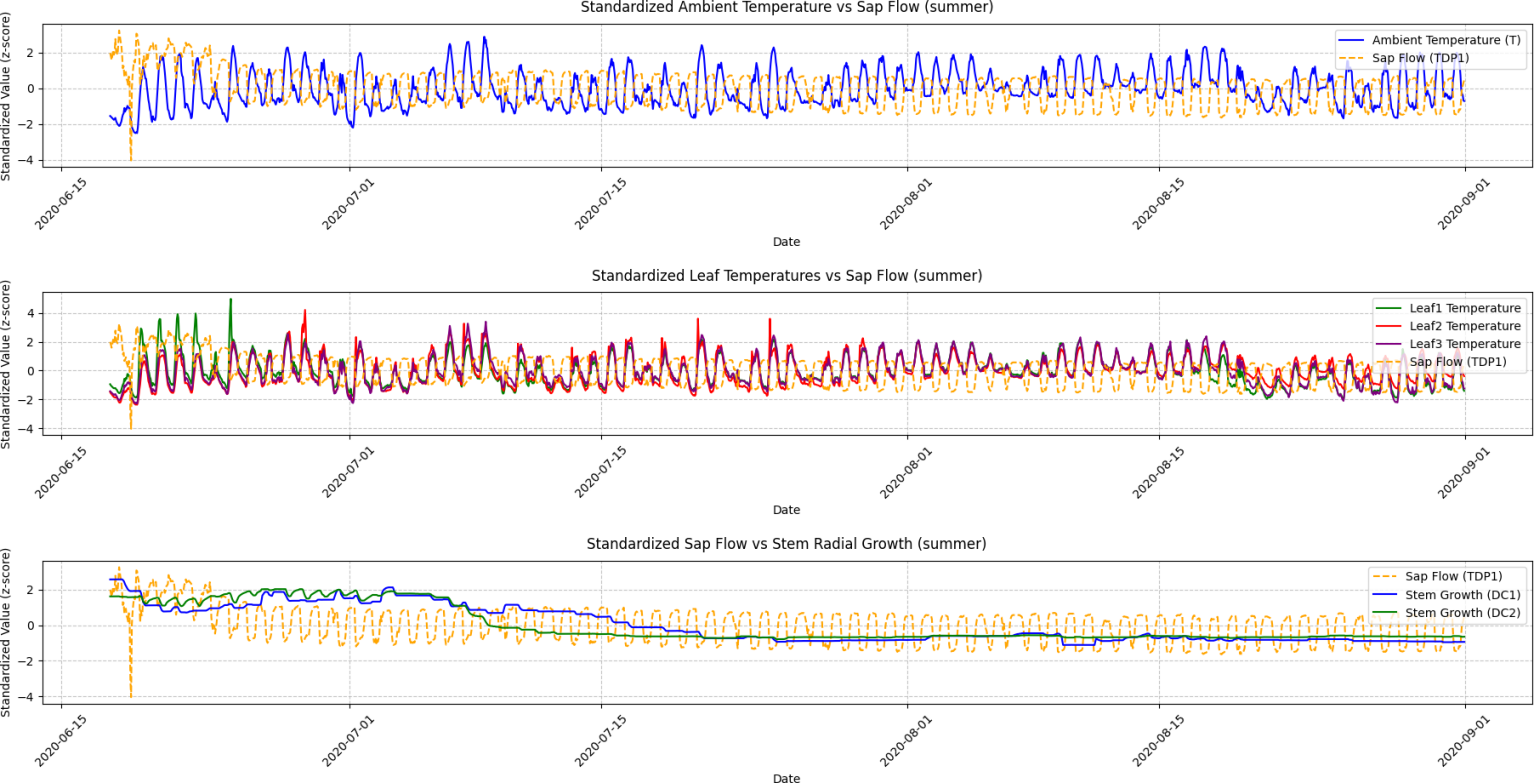
Summer seasonal variations in ambient temperature, leaf temperature, sap flow, and stem radial growth. This figure depicts the seasonal fluctuations in ambient temperature, leaf temperatures (Leaf1, Leaf2, Leaf3), sap flow (TDP1), and stem radial growth (DC1, DC2) during the summer season. The Granger Causality analysis shows a strong causal relationship between sap flow and stem radial growth, with sap flow leading growth in DC2. The figure highlights the synchronized growth patterns driven by high temperatures and abundant sunlight during this active growing season.

**Figure 6:**
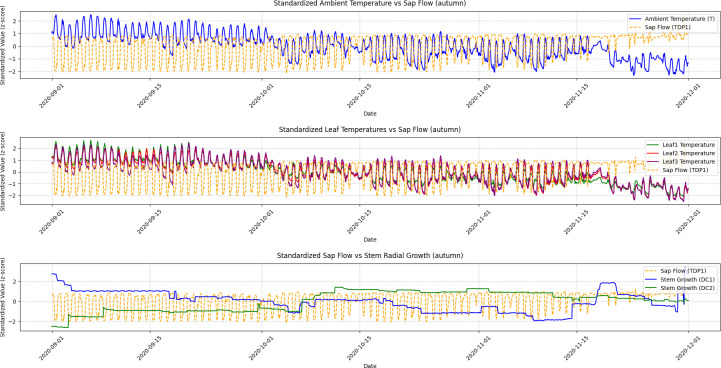
Autumn seasonal variations in ambient temperature, leaf temperature, sap flow, and stem radial growth. This figure presents the seasonal variations in ambient temperature, leaf temperatures (Leaf1, Leaf2, Leaf3), sap flow (TDP1), and stem radial growth (DC1, DC2) during autumn. Granger Causality analysis reveals that the causal relationship between sap flow and stem radial growth weakens as temperatures decline and grapevine growth slows. The reduction in sap flow and leaf temperature reflects the transition towards dormancy.

**Figure 7:**
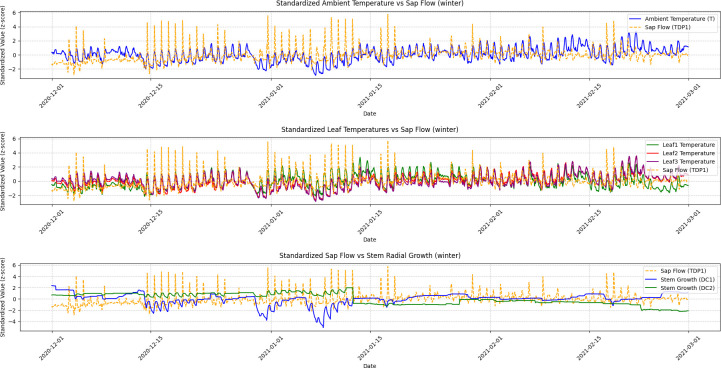
Winter seasonal variations in ambient temperature, leaf temperature, sap flow, and stem radial growth. This figure illustrates the fluctuations in ambient temperature, leaf temperatures (Leaf1, Leaf2, Leaf3), sap flow (TDP1), and stem radial growth (DC1, DC2) during the winter season. Granger Causality analysis shows minimal causal effect between sap flow and stem radial growth, reflecting the grapevine’s dormancy phase. The figure demonstrates how environmental conditions during winter lead to reduced physiological activity in the plant, with weakened correlations between growth and sap flow.

**Figure 8:**
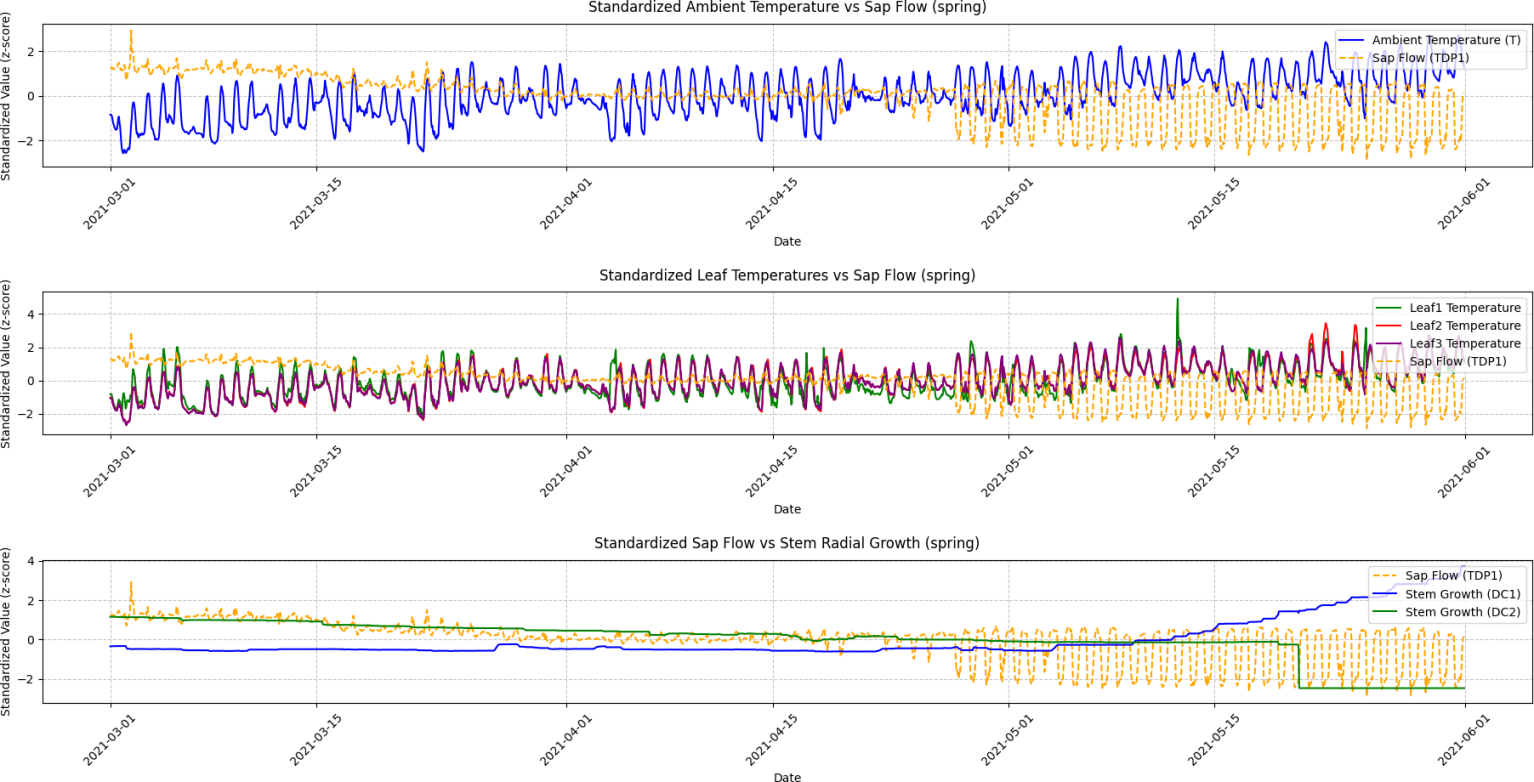
Spring seasonal variations in ambient temperature, leaf temperature, sap flow, and stem radial growth. This figure shows the fluctuations in ambient temperature, leaf temperatures (Leaf1, Leaf2, Leaf3), sap flow (TDP1), and stem radial growth (DC1, DC2) during the spring season. The results of Granger Causality analysis are presented, demonstrating that sap flow leads to changes in stem radial growth (DC2) as temperatures begin to rise. This interaction reflects the grapevine’s recovery from dormancy and the increasing influence of temperature on physiological responses.

A Granger Causality Test was performed to analyze the temporal relationships, and the visualization results illustrating these causal dynamics are presented in **Figures 5**, **6**, **7**, and **8**.

Summer: Sap flow (TDP1) had a significantly stronger causal effect on DC2 compared to other seasons. The Granger Causality test indicated a strong cause-effect relationship between sap flow and grapevine growth. The time series in **Figure 2** (Summer) clearly shows that the increases in sap flow precede the growth of DC2. The ADF test for DC1 and DC2 in the summer season (p-values < 0.05) confirmed the presence of significant stationarity after differencing, indicating that the seasonal effects were stronger and more consistent during this time. This aligns with the visual patterns in **Figure 3**, where sap flow and DC2 follow a similar trend, with sap flow leading growth in the grapevine.

Autumn: The causal relationship between sap flow (TDP1) and DC2 remained significant, but the effect was less pronounced compared to summer. The ADF test for DC2 (p-value = 0.5084) indicated that it was initially non-stationary, but after differencing, it became stationary (p-value = 0.0000). The time series in **Figure 4** (Autumn) shows moderate fluctuations in both sap flow and DC2, with sap flow continuing to lead DC2. However, the amplitude of fluctuations was smaller in the autumn, reflecting a reduction in the rate of growth compared to the summer. This seasonal shift is also captured by the ADF results, indicating less variability and a more stable relationship between sap flow and DC2 during this period.

Winter: The causal relationship between sap flow (TDP1) and DC2 was weakest, with sap flow showing minimal effect on DC2. The ADF test for DC2 (p-value = 0.6506) indicated that DC2 was non-stationary, but after differencing, it became stationary (p-value = 0.0000). The time series in **Figure 5** (Winter) shows that both sap flow and DC2 exhibit low variability and smaller fluctuations, reflecting the dormant phase of grapevine growth. The lack of significant fluctuations in sap flow during winter explains the weaker causal relationship observed. This is consistent with the general slowdown in physiological processes during the winter months.

Spring: During the spring season, sap flow (TDP1) and grapevine diameter (DC2) exhibited a noticeable temporal relationship, where increases in sap flow were followed by changes in DC2. The Granger Causality test showed that sap flow (TDP1) Granger caused changes in DC2, although the causal effect was weaker compared to other seasons. This is reflected in the lower amplitude of fluctuations in both variables during spring, as seen in **Figure 1** (Spring). The ADF test results for DC2 in spring (p-value = 0.6506) also indicated that DC2 was non-stationary, requiring differencing to make it stationary before the causality analysis. Once differenced, DC2 showed a significant p-value (0.0000), confirming the stationarity and further supporting the causal analysis.

The Granger Causality test revealed that sap flow (TDP1) consistently Granger caused changes in grapevine diameter (DC2) throughout the year. However, the strength of this causal relationship varied seasonally. The summer season exhibited the strongest causal effect, while the spring and autumn seasons showed moderate relationships. Winter, as expected, had the weakest relationship, reflecting the slower metabolic activity of the grapevine during dormancy. The ADF test results further confirmed the seasonal differences in stationarity, with the strongest variability observed in the summer and weaker fluctuations in the winter.

### 3.5 Comparison of predictive model performance

**Figure 9:**
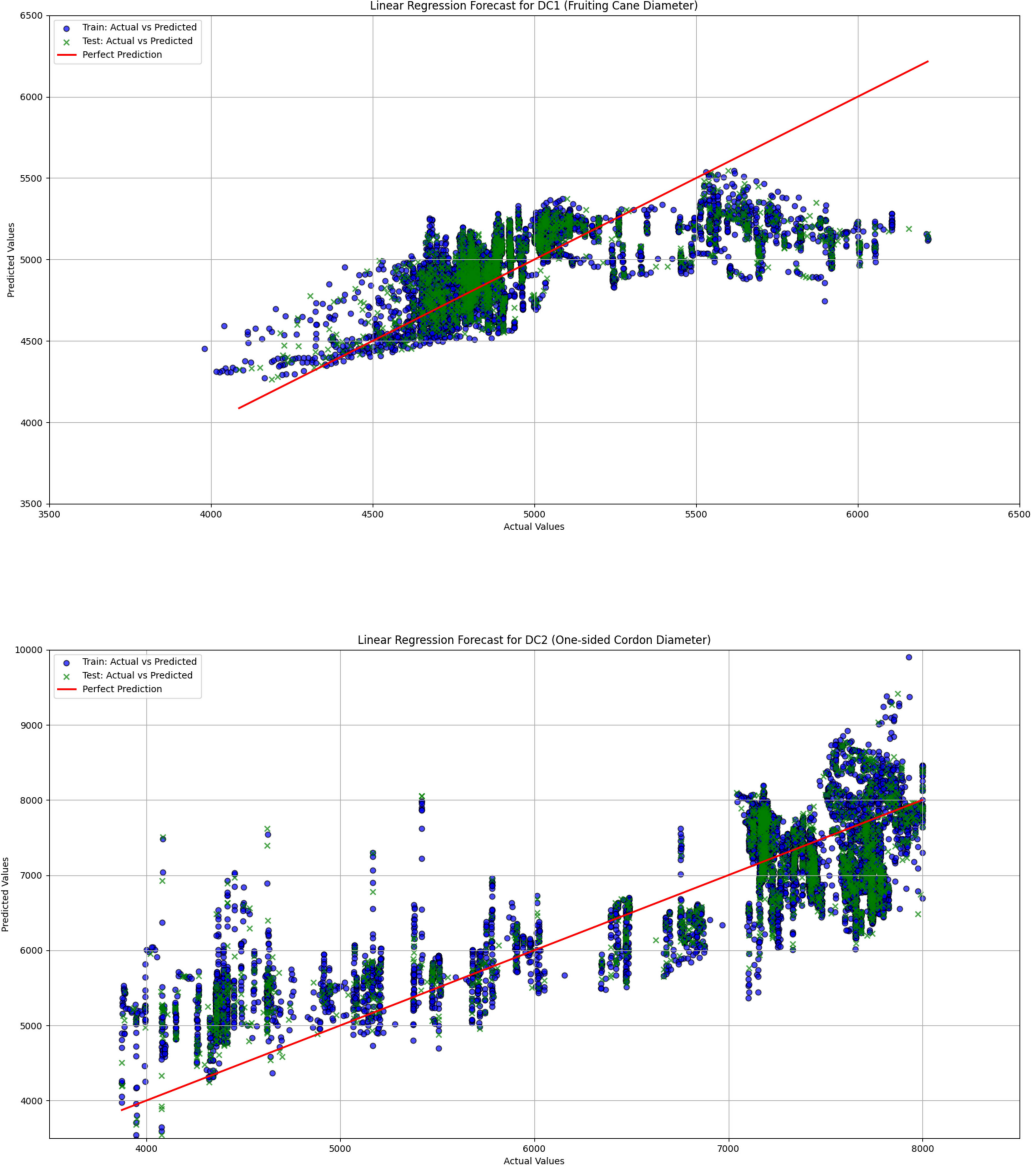
The comparison of actual and predicted values for both fruiting cane diameter (DC1) and one-sided cordon diameter (DC2) is shown using a linear regression model. This figure presents the performance of the model in predicting the growth of grapevine diameters, highlighting the correlation between observed and predicted values.

**Figure 10:**
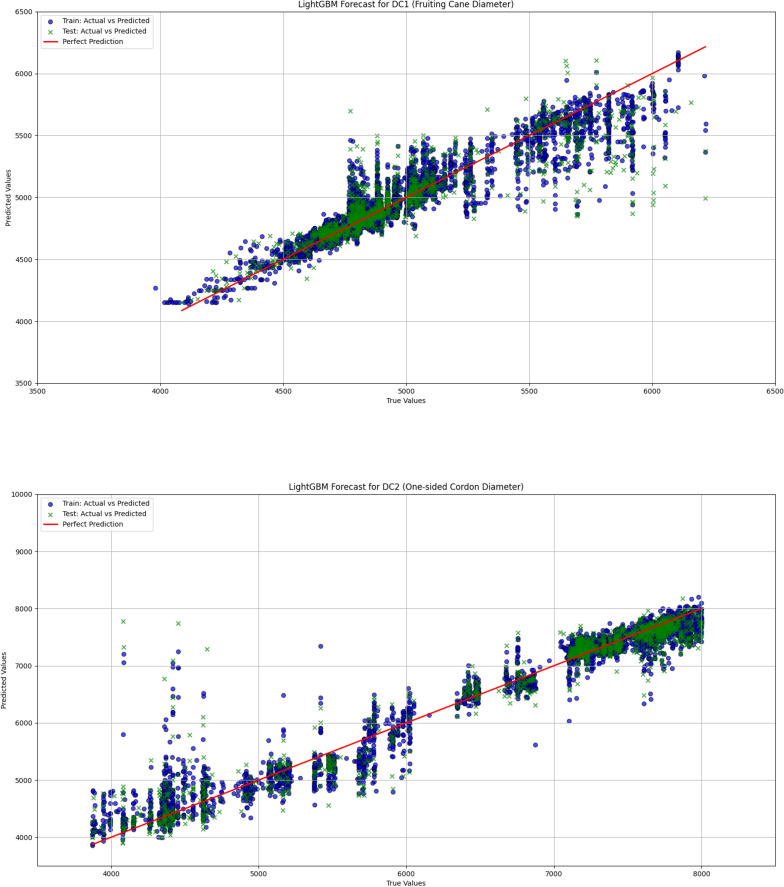
The comparison between actual and predicted values for both fruiting cane diameter (dc1) and one-sided cordon diameter (DC2) is shown using the LightGBM model. This figure illustrates the model’s performance in predicting grapevine diameter, emphasizing the accuracy and correlation between observed and predicted values for both DC1 and DC2.

**Figure 11:**
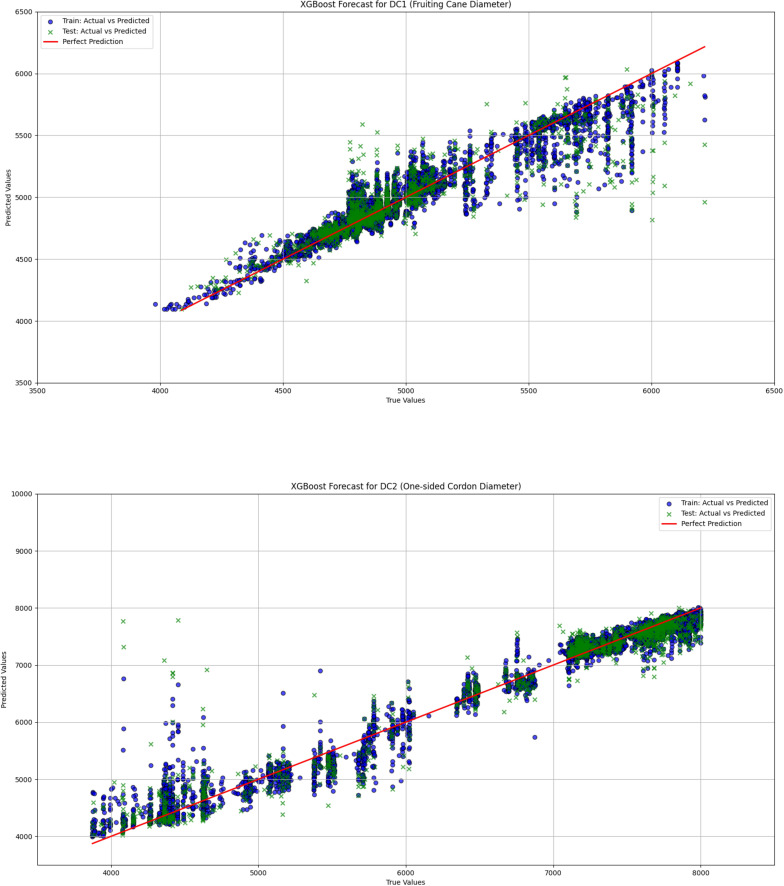
The comparison between actual and predicted values for both fruiting cane diameter (DC1) and one-sided cordon diameter (DC2) is shown using the XGBoost model. This figure highlights the model’s effectiveness in predicting grapevine diameters, showcasing the accuracy and correlation between the observed and predicted values for DC1 and DC2.

In grapevine growth prediction, the tree-based ensemble models LightGBM and XGBoost effectively captured nonlinear relationships in the data and providing high-precision predictions. To evaluate the performance of these two models, this study uses a linear regression model as a benchmark for comparative analysis, aiming to identify the most suitable model for predicting grapevine diameter. The dataset was divided into training and validation sets in an 80:20 ratio. The hyperparameters for the LightGBM and XGBoost models are shown in **Tables 1** and **2**, respectively. The input features for the models include Leaf1, Leaf2, Leaf3, TDP1, and T, with the output variables being the diameters DC1 and DC2. Through this comparative analysis, the study aims to provide more accurate predictions and decision support for grapevine management. The correlation analysis results for linear regression, LightGBM, and XGBoost are visualized in **Figures 9**, **10**, and **11**, respectively, while the detailed analysis for each model is presented in **Table 3**.

**Table 1:** LightGBM model hyperparameters.

Hyperparameter	Value
Objective	Regression
Metric	MSE, RMSE, MAE, MAPE, R²
Boosting Type	Gradient Boosting Decision Tree
Num Leaves	31
Learning Rate	0.1
Feature Fraction	0.9
Seed	42
Number of Rounds	200

**Table 2:** XGBoost model hyperparameters.

Hyperparameter	Value
Objective	Regression
Metric	MSE, RMSE, MAE, MAPE, R²
Max Depth	6
Learning Rate	0.1
Subsample	0.8
Colsample Bytree	0.8
Seed	42
Number of Rounds	200

**Table 3:** Comparison of model (Linear regression, LightGBM, and XGBoost) results (MSE, RMSE, MAE, MAPE, R²).

Model	MSE	RMSE	MAE	MAPE	R²
Linear Regression Forecast for DC1	66439.081	257.765	179.822	3.483	0.422
Linear Regression Forecast for DC2	440140.594	663.436	535.191	8.463	0.670
LightGBM Forecast for DC1	28579.034	169.053	92.714	1.785	0.751
LightGBM Forecast for DC2	93265.304	305.393	169.834	2.827	0.930
XGBoost Forecast for DC1	28303.740	168.237	91.772	1.769	0.753
XGBoost Forecast for DC2	90117.818	300.196	163.289	2.745	0.933

The linear regression model performed poorly in predicting both DC1 and DC2, especially in the prediction of DC2, where the R² value was only 0.670, significantly lower than those of the LightGBM and XGBoost models (0.930 and 0.933, respectively). This indicates substantial limitations in the ability of the linear regression model to capture complex relationships in the data. Furthermore, the Mean Squared Error (MSE) and Root Mean Squared Error (RMSE) for DC1 and DC2 were both relatively high for the linear regression model, at 66439.081 and 257.765 (DC1) and 440140.594 and 663.436 (DC2), indicating large prediction errors. Particularly in the prediction of DC2, the linear regression model’s Mean Absolute Error (MAE) and Mean Absolute Percentage Error (MAPE) were 535.191 and 8.463%, further demonstrating the low prediction accuracy of this model on this dataset. In contrast, both the LightGBM and XGBoost models exhibited significantly lower MSE, RMSE, MAE, and MAPE for both DC1 and DC2, indicating higher prediction accuracy and better fitting performance. These results suggest that tree-based ensemble learning methods outperform traditional linear regression models when handling complex datasets. These results support a plant-based, WUE-oriented scheduling approach in which sap flow and leaf/diameter signals provide the primary predictors, consistent with prior sap-flow–WUE analyses.

### 3.6 Seasonal forecasting of DC1 and DC2 using the prophet model

**Figure 12:**
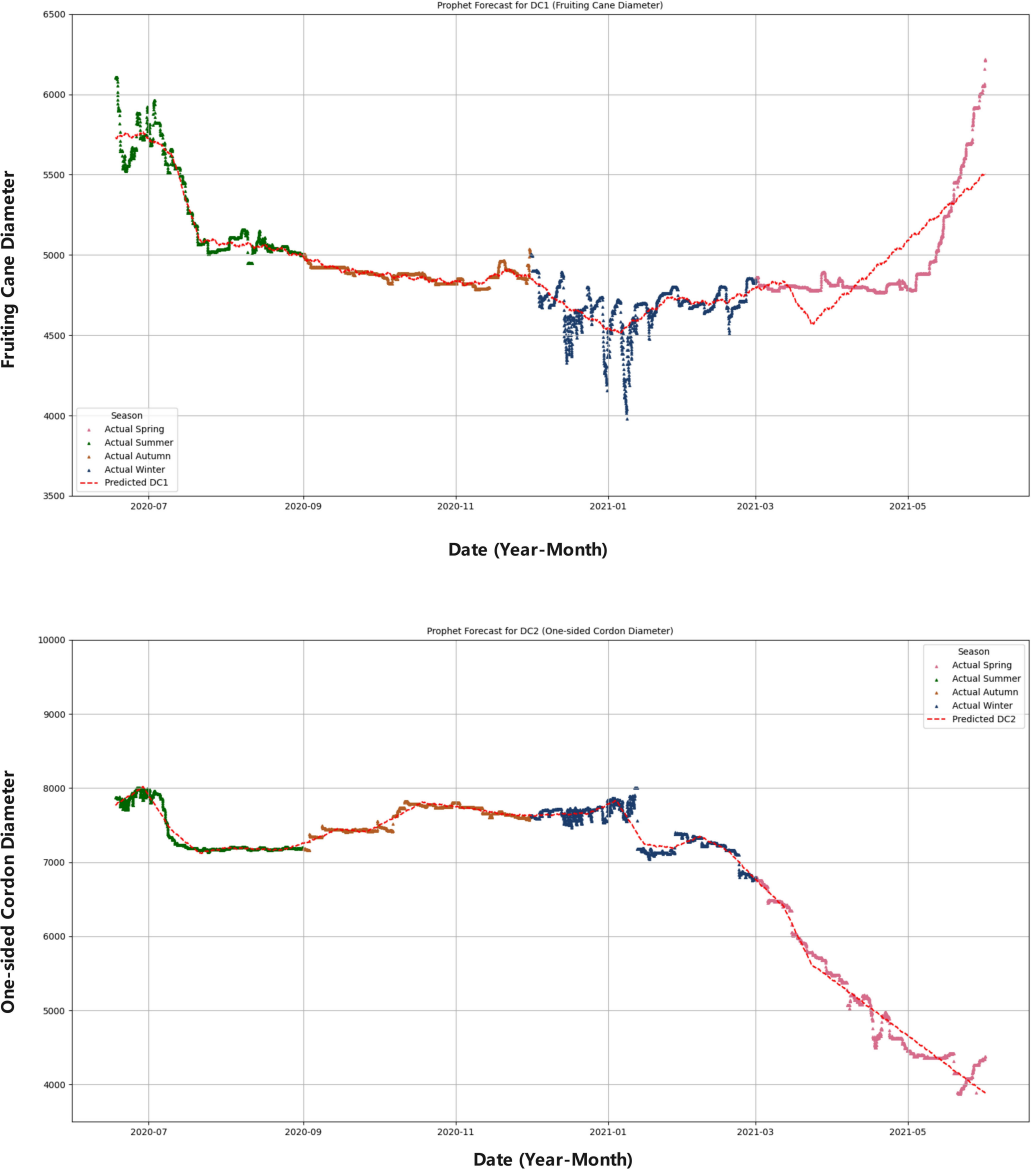
Seasonal comparison of DC1 and DC2 predictions. This composite figure compares the predicted values of fruiting cane diameter (DC1) and one-sided cordon diameter (DC2) with their respective actual seasonal data. The figure uses different colors to represent each season: spring (pink), summer (green), autumn (orange), and winter (blue). The red dashed line indicates the predicted values for both DC1 and DC2, allowing for an easy comparison between the model’s predictions and the observed seasonal growth patterns.

The performance evaluation of the LightGBM and XGBoost models demonstrated that these tree-based ensemble learning methods performed very well in capturing the nonlinear relationships in grapevine diameter predictions. However, these models did not account for seasonal information. The Prophet model has shown significant advantages in capturing the seasonal fluctuations of grapevine growth. Therefore, to conduct seasonal predictions, this model was used in the psresnet study to further analyze the prediction capabilities for DC1 and DC2. The hyperparameters for the Prophet model are listed in **Table 4**. The correlation analysis results are shown in the **Figures 12**, and the detailed analysis results are presented in the **Table 5**.

**Table 4:** Prophet model hyperparameter.

Hyperparameter	Value
Seasonality	additive
Metric	MSE, RMSE, MAE, MAPE, R²
Seasonality Prior Scale	10
Holidays Prior Scale	10
Changepoint Prior Scale	0.05
Periodicity	365

**Table 5:** Prophet model seasonal training results comparison (MSE, RMSE, MAE, MAPE, R²).

Model	Season	MSE	RMSE	MAE	MAPE	R²
Prophet Forecast for DC1	Summer	7105.390	84.293	57.009	1.043	0.928
	Autumn	815.682	28.560	20.494	0.420	0.623
	Winter	11493.433	107.207	77.679	1.690	0.393
	Spring	41883.498	204.655	161.690	3.182	0.629
	Overall	15782.446	125.628	80.461	1.612	0.847
Prophet Forecast for DC2	Summer	4689.045	68.477	44.460	0.590	0.950
	Autumn	2324.675	48.215	35.708	0.472	0.913
	Winter	12237.328	110.622	74.878	1.005	0.861
	Spring	23889.474	154.562	119.491	2.510	0.965
	Overall	11093.525	105.326	69.853	1.174	0.991

The Prophet model demonstrated significant seasonal fluctuations in the predictions for DC1 and DC2. For DC1, the model showed higher prediction accuracy in summer, with an MAE of 57.009 and an R² of 0.928, while the prediction error was larger in spring, with an MAE of 161.690 and an R² of 0.629. In contrast, the prediction for DC2 was more stable, particularly during spring and summer, with MAEs of 119.491 and 44.460 and R² values of 0.965 and 0.950, respectively. In comparison, the LSTM and Bi-LSTM models, as shown in the **Table 6**, performed worse than the Prophet model. For DC1, the LSTM model achieved an R² of 0.818 and the Bi-LSTM model achieved an R² of 0.805, indicating weaker performance in capturing seasonal growth compared to Prophet. Similarly, for DC2, the LSTM model reached an R² of 0.919, and the Bi-LSTM model achieved an R² of 0.910, still falling short of the exceptional R² of 0.991 from the Prophet model. This discrepancy highlights the lack of explicit seasonality handling in the LSTM and Bi-LSTM structures, which contributed to their weaker performance in predicting seasonal growth.

**Table 6:** Model seasonal training results comparison (MSE, RMSE, MAE, MAPE, R²).

Model	MSE	RMSE	MAE	MAPE	R²
Prophet Forecast for DC1	15782.446	125.628	80.461	1.612	0.847
Prophet Forecast for DC2	11093.525	105.326	69.853	1.174	0.991
LSTM Forecast for DC1	16532.431	128.072	85.462	1.740	0.818
LSTM Forecast for DC2	11856.789	107.259	72.981	1.347	0.919
Bi-LSTM Forecast for DC1	17987.562	130.384	88.672	1.790	0.805
Bi-LSTM Forecast for DC2	12992.644	110.454	74.765	1.380	0.910

The Prophet model showed significant seasonal fluctuations in the predictions for both DC1 and DC2. The results for DC1, with a model R² of 0.847 overall, and for DC2, with an exceptional R² of 0.991, suggest that while the Prophet model effectively captured seasonal growth patterns, DC1 was more influenced by environmental changes, particularly during the spring and winter months. In comparison, the LSTM and Bi-LSTM models, which performed worse than the Prophet model, showed weaker performance in predicting seasonal growth due to the lack of explicit seasonality handling in their structure.

While this study primarily focused on capturing seasonal fluctuations in grapevine growth through the Prophet model, it is important to acknowledge that there are other potential explanations for variations in grapevine growth dynamics that were not explicitly explored in this analysis. For example, inverse sap flow could play a role in altering the growth patterns, particularly during night-time when transpiration is low but the grapevine may still experience water transport through reverse flow. Additionally, night-time fluxes, which are influenced by the plant’s internal water balance during non-transpirational periods, could contribute to the overall water transport system in ways not fully captured in this study.

Furthermore, cold-stress induced xylem refilling is another potential factor that may affect water transport, particularly during the winter months when the grapevine is in dormancy. This phenomenon could introduce fluctuations in sap flow that are not solely driven by environmental temperature but by internal physiological mechanisms in response to freezing temperatures.

Future studies could consider incorporating these alternative explanations to provide a more comprehensive understanding of the factors influencing grapevine growth and water transport across different seasons.

## 4 Discussion

### 4.1 Model performance

The comparison of the prediction results from LightGBM, XGBoost, and linear regression models demonstrated that tree-based ensemble learning methods (LightGBM and XGBoost) had significant advantages in addressing the problem of grapevine diameter prediction. The linear regression model, due to its limitations in linear assumptions, was unable to effectively capture the nonlinear relationships within the data, leading to poorer performance in predicting both DC1 and DC2. Particularly in the prediction of DC2, the R² value of the linear regression model was only 0.670, which was far lower than the R² values of LightGBM and XGBoost (0.933 and 0.930, respectively). This indicates that the linear regression model has evident limitations when handling complex datasets. This interpretation is consistent with prior solar-greenhouse evidence that links sap-flow dynamics to physio-environmental drivers and water-use efficiency (WUE), and treats diurnal hysteresis as a decision-relevant diagnostic (Wei et al., 2020).

In contrast, LightGBM and XGBoost models, by integrating multiple decision trees, were able to better capture the nonlinear relationships within the data, resulting in higher prediction accuracy for both DC1 and DC2. Specifically, the XGBoost model achieved an R² value of 0.933 for DC2, showcasing its robust capability in handling complex data. Furthermore, the MSE, RMSE, MAE, and MAPE values for both LightGBM and XGBoost were significantly lower than those of the linear regression model, further validating their superiority in grapevine diameter prediction. In practice, ensemble predictions of DC1/DC2 from plant signals can be coupled to season-specific thresholds to drive irrigation timing and amounts in a decision-oriented manner.

### 4.2 Seasonal discussion based on the prophet model

The Prophet model demonstrated excellent performance in capturing the seasonal variations in grapevine diameter. Through the seasonal forecasting of DC1 and DC2, it was found that the Prophet model could effectively reflect the seasonal fluctuations in grapevine diameter. Notably, for DC2, the prediction accuracy was high, with an R² value close to 1 (0.991), indicating that the model was able to predict grapevine diameter changes with high precision. In contrast, the prediction accuracy for DC1 was slightly lower, with an R² value of 0.847, which could be attributed to the greater sensitivity of DC1 to environmental factors.

As the DC1 is more complex in terms of growth changes, especially in the spring and autumn, fluctuations in external environmental factors tend to cause significant variations in growth, making the prediction more challenging. In contrast, the DC2 exhibits relatively stable growth, less affected by environmental fluctuations, thus allowing for more accurate predictions using the Prophet model. The relative stability of DC2 suggests that the cordon better represents a whole-axis transport baseline, whereas DC1 (fruiting cane) is more sensitive to short-term microclimate and growth transitions.

The lower performance in spring and winter likely reflects dormancy and transition stages, where physiological noise and low signal variance challenge Prophet’s seasonality assumptions. During winter, the grapevine enters a dormant phase, with minimal metabolic activity and negligible changes in DC1, leading to low variance in the data. This lack of growth variability makes it difficult for the Prophet model to detect meaningful patterns and accurately forecast DC1. Similarly, in spring, the rapid transition from dormancy to active growth creates more fluctuating environmental conditions, including temperature and moisture changes, which further complicate the growth dynamics of DC1. These periods of physiological noise and reduced predictability make it harder for Prophet’s model to maintain high accuracy, as it relies on seasonal patterns and stable trends to make predictions.

### 4.3 Model application and decision-making

Using five variables (Leaf1, Leaf2, Leaf3, TDP1, and T), the LightGBM and XGBoost models were able to predict grapevine diameter changes with good accuracy. These models considered the multifaceted impact of environmental factors on grapevine growth, providing valuable decision support for vineyard management. For example, based on input data such as leaf temperature, trunk sap flow, and environmental temperature, the models can predict grapevine diameter trends under varying temperature conditions, allowing for adjustments in irrigation, fertilization, and water transport strategies to ensure adequate water and nutrient supply during growth, thus mitigating growth suppression caused by extreme environmental changes. For warm seasons and spring, an actionable composite trigger is: (i) an upper-canopy leaf-temperature rise (Leaf2 or Leaf3) relative to air, together with (ii) a same-day drop of TDP1 below its morning baseline (e.g., within-day 30th percentile). This joint plant-based cue targets physiological stress beyond meteorological warming and aligns with a WUE-oriented perspective.

Moreover, these models can predict changes in grapevine diameter during different growth stages and provide precise guidance for pruning and support management. During the rapid growth phase, the models can help adjust pruning timing and support measures to promote healthy grapevine growth. In extreme temperature conditions, the models can forecast diameter trends, enabling timely protective measures to minimize environmental stress on the grapevines. Thresholds are phenology-specific: cane-based dynamics (DC1) provide early warning during spring recovery, whereas cordon-based confirmation (DC2) stabilizes decisions in summer.

Future work will integrate additional environmental variables including soil moisture, humidity, photosynthetically active radiation (PAR), and light intensity. These additions will enhance model robustness and ecological relevance. Future deployment should benchmark decision thresholds against WUE metrics to enable unified evaluation across cultivars and training systems.

### 4.4 Comparative discussion with existing models and studies

This study’s approach using physiological sensors (e.g., sap flow sensors, branch diameter sensors) for monitoring grapevine growth shows promising results. However, it can be further contextualized by comparing it to studies using other advanced sensing technologies, such as SIF (Solar-Induced Fluorescence), NDVI (Normalized Difference Vegetation Index), and SWC (Soil Water Content) sensors, which are also used to monitor vineyard conditions.

For example, studies utilizing SIF sensors have demonstrated their ability to assess grapevine photosynthesis and provide insights into plant stress and growth dynamics (Zhao et al., 2022). Similarly, the NDVI index, a widely used vegetation index, has been employed to assess vine vigor and health, offering a more comprehensive understanding of canopy cover and growth patterns (Mazzetto et al., 2010). Moreover, SWC sensors have been utilized to monitor soil moisture content, which is critical for understanding water availability and its impact on grapevine growth (Horel and Zsigmond, 2023).

Comparing the performance of these sensors with those used in this study, such as sap flow sensors and diameter sensors, could offer valuable insights into the strengths and limitations of each technology. For example, while NDVI and SIF sensors provide a broader view of canopy health and photosynthetic activity, sap flow sensors offer more direct measurements of physiological processes such as water transport. Combining these different types of sensors could provide a more integrated and accurate model for predicting grapevine growth and health.

### 4.5 Long-term monitoring system and future prospects

While current models such as LightGBM, XGBoost, and Prophet capture environmental factors and seasonal fluctuations well, they still have room for improvement. One limitation is the lack of additional sensor data, such as soil moisture and meteorological information, which could improve model accuracy by capturing complex nonlinear relationships between environmental variables and grapevine growth.

Future studies should explore deep learning approaches, particularly time-series architectures, for more precise predictions. Expanding the research to multiple outdoor field sites with varying environmental conditions would also enhance the generalizability of the results, compared to the current single-site greenhouse experiment.

To strengthen the theoretical foundation, integrating the Soil–Plant–Atmosphere Continuum (SPAC) model would allow explicit coupling of soil moisture, plant water uptake, and atmospheric demand. This holistic framework could deepen the understanding of grapevine water transport and inform more effective irrigation strategies. Moreover, deploying field validation trials outside the plastic greenhouse would broaden applicability and provide insights into model robustness under diverse real-world conditions.

Finally, future studies should link physiological responses to yield and quality metrics, such as cluster weight, berry sugar content (Brix), and phenolic composition. This integration would bridge environmental responses with agronomic performance, providing growers with actionable insights for optimizing vineyard management.

## 5 Conclusion

This study advances a plant–sensor view of grapevine water relations by integrating trunk sap flow, organ-resolved radial growth (DC1, DC2), and leaf temperature across a full annual cycle. Three contributions emerge. First, a season-dependent sign structure was identified for the temperature–sap-flow coupling: negative in spring, summer and autumn and positive in winter, revealing predictable shifts in driver–response coordination across phenological stages. Second, organ-specific coordination was resolved, with the cordon (DC2) acting as a stable transport baseline and the fruiting cane (DC1) exhibiting higher short-term sensitivity and asynchrony during transitions. Third, time-ordering analyses showed that sap flow statistically leads diameter change in active seasons, providing a physiological basis for plant-based control.

Methodologically, the study operationalizes multi-sensor physiology for decision support. A season-aware baseline (Prophet) captured low-frequency growth rhythms, while tree-based ensembles (LightGBM, XGBoost) reproduced high-frequency nonlinear variation in DC1/DC2 with markedly lower error than a linear benchmark. Together, these components form a practical forecasting stack that converts continuous plant signals into decision-ready indicators.

In terms of application, a composite, plant-based trigger is proposed for warm seasons and spring: (i) an upper-canopy leaf-temperature rise (Leaf2/Leaf3) relative to ambient air, together with (ii) a same-day drop of trunk sap flow (TDP1) below its morning baseline (e.g., within-day 30th percentile). This joint cue targets physiological stress rather than meteorological heat alone and supports phenology-aware irrigation, in which DC1 provides early warning during spring recovery while DC2 stabilizes decisions in summer. The framework yields clear actions: advance irrigation timing under sustained trigger exceedance; relax or defer irrigation during winter dormancy when positive temperature–sap-flow coupling indicates low demand; and taper irrigation in late season as triggers abate and growth slows.

Conceptually, the findings link season-dependent coupling and organ-specific coordination to actionable management, bridging descriptive sensor traces and irrigation scheduling. Practically, the approach is compatible with existing vineyard operations and can be extended to other perennial systems that benefit from continuous monitoring.

Limitations include a single site and protected-environment setting, limited edaphic sensing, and no direct linkage to yield and quality outcomes. Future work should calibrate season-specific thresholds across cultivars and training systems in open-field conditions, incorporate soil moisture and radiation to tighten attribution, and co-evaluate decisions against water productivity and fruit quality metrics. Such extensions will generalize the thresholds and quantify operational gains, completing the path from multi-sensor physiology to scalable precision irrigation.

## Data Availability

The raw data supporting the conclusions of this article will be made available by the authors, without undue reservation.
